# Blossoms in perovskite planar X-ray detectors

**DOI:** 10.1038/s41467-024-50179-2

**Published:** 2024-07-09

**Authors:** Xiangyu Ou, Feng Gao

**Affiliations:** Department of Physics, Chemistry and Biology (IFM), Linköping University, Linköping, Sweden

**Keywords:** Materials for devices, Materials chemistry

## Abstract

Solution processable perovskites are revolutionising the research field of direct X-ray detectors. Here, the authors discuss the opportunities, challenges, and research strategies for perovskite planar X-ray detectors.

## Teaser

Solution processable perovskites have made remarkable strides in direct planar X-ray detectors, promising to reshape X-ray detector industry. Herein, we discuss prevailing challenges, research opportunities, and their synergies with cutting-edge technologies that will benefit both human society and scientific research.

X-ray imaging, a technique utilising X-rays to reveal the internal structure of matter through planar X-ray detectors, has significantly advanced scientific research and modern society. In general, indirect planar X-ray detectors convert X-rays into visible photons through scintillators, while direct planar X-ray detectors convert X-rays into charge carriers through semiconductors. With increasing needs for X-ray imaging applications, achieving lower radiation dosage and higher spatial resolution is the primary goal of next-generation planar X-ray detectors. Particularly, direct planar X-ray detectors, featuring high spatial resolution as charge carriers move along the electric field with nearly no signal crosstalk, are optimal for this ambition. However, the quest for excellent semiconductors that meet all prerequisites of X-ray detection and can be readily integrated with readout electronics for planar X-ray detectors remains a highly challenging endeavour.

## The rising star for planar X-ray detectors

As the centrepiece of direct X-ray detectors, semiconductors play a decisive role in detection performance. However, commercial amorphous selenium (α-Se) and cadmium zinc telluride (CdZnTe) suffer from either low X-ray absorption or rigorous manufacturing, presenting inherent limitations for planar X-ray detectors.

**Fig. 1:**
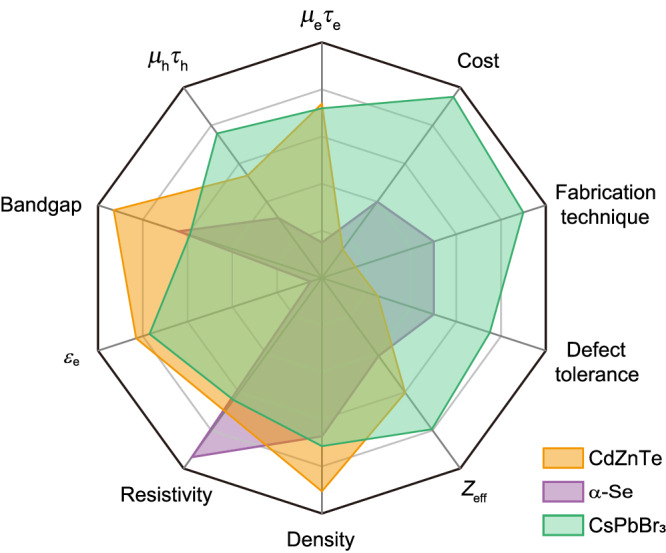
The performance radar of CdZnTe, α-Se, and CsPbBr_3_. While CdZnTe single crystals exhibit excellent optoelectronic properties for X-ray detection, their manufacturing process is harsh and costly. On the contrary, CsPbBr_3_ displays comparable performance to CdZnTe and is suitable for large-area fabrication via solution processes at a low cost. *μ*_e_*τ*_e_ and *μ*_h_*τ*_h_ represent the mobility-lifetime product of electrons and holes, respectively; *ε*_e_ represents electron-hole pair production energy; *Z*_eff_ represents the effective atomic number.

Emerging in 2009, perovskites have entirely transformed the conventional perception of high-performance semiconductors because they can be solution-processed while maintaining excellent optoelectronic properties. Remarkably, perovskite solar cells have reached a certificated efficiency of 26.15 %^1^, close to silicon solar cells (27.6 %) developed for nearly 70 years. In 2013, perovskites stepped onto the historical stage of the high-energy radiation detection field^2^, showing great promise to develop next-generation planar X-ray detectors^3^ (Fig. 1 and Table 1):

**Table 1:** Comparison of optoelectronic properties of CdZnTe, a-Se, and CsPbBr_3_

Materials	CdZnTe	α-Se	CsPbBr_3_
*μ*_e_*τ*_e_ (cm^2^ V^−1^)	4 × 10^−3^	5 × 10^−9^	2.05 × 10^−3^
*μ*_h_*τ*_h_ (cm^2^ V^−1^)	3.76 × 10^−5^	1.4×10^−7^	5.79 × 10^−3^
Bandgap (eV)	1.57	2.15	2.29
*ε*_e_ (eV)	4.64	40-50	5.3
Resistivity (Ω cm)	3 × 10^10^	4 × 10^12^	4 × 10^9^
Defect tolerance	Low	Medium	High
*Z*_eff_	50	34	65.9
Density (g cm^−3^)	5.8	4.3	4.57
Fabrication technique	Molten growth	Thermal evaporation	Solution process
Cost	Expensive	Medium	Low cost
References	^16–18^	^19–22^	^4,8^

Governed by the photoelectric effect (*Z*^3^*E*^−3^, where *Z* is the atomic number and *E* is the energy of the incident photon), high-*Z* perovskites exhibit strong X-ray stopping power to attenuate 90% of incident X-rays with a low thickness. Long carrier diffusion lengths ensure efficient charge carrier transport and extraction in thick active layers with a thickness of several hundred microns. The bandgaps, ranging from 1.3 to 2.3 eV depending on the chemical composition, facilitate an optimal balance between dark current levels and charge carrier numbers. Additionally, the solution processability makes perovskites unprecedented candidates for planar X-ray detectors fabricated through a wide range of low-cost and low-energy consumption techniques, which are hardly possible for conventional semiconductors^4^. High defect tolerance further endows perovskites with self-healing capability and good radiation hardness, which are essential for the long-term stability of X-ray detectors^5^.

## State-of-the-art perovskite planar X-ray detectors

In 2017, a groundbreaking research conducted by Samsung successfully integrated methylammonium lead iodide perovskites onto amorphous silicon (α-Si) thin-film transistor (TFT) backplanes through blade coating, followed by the validation of X-ray imaging capability. This pioneering work ignited the research boom on planar X-ray detectors based on perovskites^6^.

While previous research mainly concentrated on the development of perovskite planar X-ray detector based on α-Si TFT backplane with low electron mobility, a recent research has demonstrated the feasibility of integrating caesium lead bromide (CsPbBr_3_) perovskites onto a complementary metal oxide semiconductor (CMOS) array (72 × 72; pixel size: 83.2 microns) through layer-by-layer screen printing^7^. Alternatively, CMOS built on single-crystal silicon with excellent electron mobility and smaller pixels provides a prospective option for high-performance planar X-ray detectors, enabling faster readout speed and higher spatial resolution over the TFT technique. Hence, CsPbBr_3_ CMOS X-ray detectors achieve an unprecedented spatial resolution (5 line pair mm^−1^) under low dosage (260 nGy) and a fast readout speed of 300 frames per second (fps). Although the pixel of the proposed CMOS array is 83.2 microns with a relatively limited active region in this research, the state-of-the-art CMOS has reached a pixel size of 5 microns with a larger active area, allowing for further enhancing the capabilities of the perovskite CMOS planar X-ray detectors. Notably, thickness modulation is required to mitigate the signal crosstalk upon coupling to a CMOS array with smaller pixels since horizontal charge crosstalk is severe in this case.

Simultaneously, a research team from Huazhong University of Science and Technology integrated CsPbBr_3_ perovskite single crystals onto CMOS chips through anisotropic conductive bonding, a well-established technology in the flat-panel screen industry^8^. CsPbBr_3_ CMOS X-ray detectors realise intelligent X-ray imaging with the assistance of in-sensor computing technology, achieving precise edge extraction and a ca. 50% data compression for energy saving. Moreover, these intelligent X-ray detectors could be programmed and trained to carry out image recognition with an accuracy of 100%.

## Opportunities

The solution processability offers extraordinary possibilities for perovskites to incorporate with organic polymer substrates through embedding or surface deposition to fabricate flexible planar X-ray detectors^9^. As flexible planar X-ray detectors can be intentionally shaped to fit the surface geometry of the objects, they present higher spatial resolution, reduced radiation exposure, and improved user comfort, especially suitable for mammography without breast compression, painless dental X-rays, and trauma radiography. What’s more, the lightweight and portable flexible X-ray detectors expand indoor medical diagnostics to outdoor emergencies, greatly scaling medical efficiency and accessibility.

Thanks to the high defect tolerance of perovskites, polycrystalline perovskites demonstrate comparable performance to single-crystal perovskites. In addition, the larger bandgap of perovskites compared to silicon contributes to a lower dark count rate at room temperature. These properties create excellent possibilities to develop large-area photon-counting perovskite planar X-ray detectors featuring intrinsic energy spectral sensitivity, low electronic noise, and reduced radiation exposure, holding great promise for high-end photon-counting computed tomography^10^.

## Challenges and strategies

**Fig. 2:**
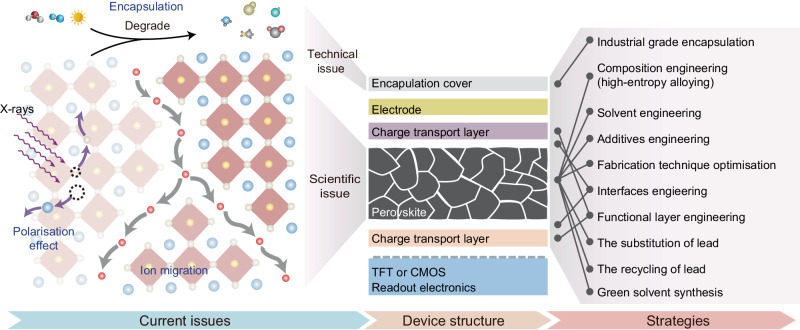
The challenges and research strategies for perovskite planar X-ray detectors. The current issues (ion migration, polarisation, encapsulation) existing in perovskite planar X-ray detectors and the feasible strategies for future research. The perovskites without encapsulation tend to decompose to several by-products at ambient conditions (water, oxygen, and lights). The grain boundaries and defects provide migration channels (grey arrows) for mobile ions (red balls). The interaction of X-rays and perovskite lattice may produce defects (dotted circles and purple arrows), inducing polarisation under bias.

Despite impressive achievements alongside the promising expectations highlighting the superiorities of perovskite planar X-ray detectors, significant barriers remain between laboratory research and commercialisation (Fig. 2).

Intrinsic ion migration, originating from lattice defects and numerous grain boundaries in thick active layers (400–1200 microns), is considered as the primary factor accounting for high dark current levels (10^−8^–10^−5^ A cm^−2^) and serious current drift in perovskite planar X-ray detectors, leading to unsatisfactory signal-to-noise ratio, compromised linear dynamic range, and degraded operation stability. Although previous studies were obsessed with increasing the detection sensitivity, a dark current below 10^−9^ A cm^−2^ is required for the integration of perovskites onto commercial readout integrated circuits, as an excessive dark current will fully charge the storage capacitor. Therefore, it is essential to suppress the dark current and current drift by inhibiting the ion migration. Solvent engineering, additives engineering, interface engineering, and fabrication technique optimisation provide effective strategies to reduce grain boundaries and defect density. In addition, elaborately modulating the A-site and B-site composition of perovskites through high-entropy alloying helps to reduce the defect density for ultrasensitive and stable X-ray detection^11^. After the fabrication of perovskite thick layers, the remaining pinholes and grain boundaries could be partially mitigated by solid-state hot pressing^12^. Furthermore, the introduction of charge blocking layers provides an additional approach to suppress the dark current by elevating the injection energy barriers. Overall, a comprehensive optimisation of the perovskite in bulks, surfaces, interfaces, and device architecture^13^ is imperative to mitigate ion migration in perovskite X-ray detectors.

X-ray detectors operating under constant X-ray flux ensure reliable dynamic X-ray imaging for medical and industrial purposes. Unfortunately, continuous external electric fields and high-intensity X-ray flux are likely to induce the polarisation in perovskite X-ray detectors, leading to abnormal photocurrent lag detrimental to the readout speed and long-term stability. Ion migration is one of the possible reasons causing polarisation due to the disruption of the electric field in perovskites by ion redistribution. Besides, the interaction between high-energy X-rays and perovskite lattice may lead to the dislocation of the atoms, introducing traps that capture the charge carriers. A recent research indicated that the polarisation behaviour of perovskite X-ray detectors is closely associated with trap density within perovskites, with lower trap density contributing to the mitigated polarisation effect^14^. Until now, the polarisation mechanism in perovskite X-ray detectors remains unclear, holistic characterisations together with theoretical calculations are required to have a deep understanding on the radiation- and bias-induced polarisation in perovskite X-ray detectors.

At present, most laboratory-based perovskite planar X-ray detectors without appropriate encapsulation are tested under ambient conditions. This practice raises concerns on long-term stability as perovskites are highly sensitive to water and oxygen, resulting in irreversible deterioration. Even worse, exposure to ambient light initiates complex photochemical reactions between perovskites and oxygen (Fig. 2), accelerating the degradation. Considering that industrialised conversions impose stringent requirements (lifecycle expectancy: 7 years; exposure levels: 21,000 times), it is necessary to sufficiently encapsulate the perovskite planar X-ray detectors to completely separate the water and oxygen.

In addition to the advancement of laboratory-based perovskite X-ray planar detectors, establishing standardised procedures for evaluating their imaging performance is of critical importance for future applications. Currently, modulation transfer function (MTF) is widely employed to assess the resolving capability of perovskite planar X-ray detectors. While MTF is an important indicator of the imaging system’s spatial resolution, the X-ray source and system noise also affect the image quality. Therefore, detective quantum efficiency (DQE), ranging from 0 to 1, is introduced to characterise the efficiency of the imaging system to convert X-rays into digital images, with a higher DQE corresponding to a better overall imaging performance^15^. By implementing standardised procedures to measure the MTF and DQE, perovskite planar X-ray detectors are anticipated to achieve fast development, reliable performance tracking, and broader market acceptance.

Indeed, the research on perovskite planar X-ray detectors is still in its early stages. Nevertheless, we are optimistic that the challenges faced by perovskite X-ray detectors can be overcome by borrowing successful experiences from other perovskite optoelectronic devices. At the same time, the employment of lead in perovskites imposes potential risks to the environment and humans. The substitution of lead, lead recycling, and green solvent synthesis are potential future research objectives for environmental protection.

## Summary

Exploiting novel semiconductors for next-generation planar X-ray detectors with the ambition to achieve low radiation exposure and high sensitivity is highly demanded. Solution-processed perovskites are promising candidates due to their high X-ray stopping energy, long charge carrier diffusion lengths, high defect tolerance, and cost-efficiency fabrication over large areas. In particular, the unique solution processability and high defect tolerance of perovskites allow for the incorporation of perovskite planar X-ray detectors with cutting-edge technologies, such as flexible electronics and photon-counting techniques, to boost X-ray imaging capabilities. However, to realise reliable perovskite planar X-ray detectors, critical challenges, including ion migration, polarisation effect, and encapsulation issues, are required to be comprehensively investigated and well addressed. In addition, standard procedures of performance evaluation should be established. By addressing these challenges, we expect that perovskite planar X-ray detectors will become a game changer in the X-ray industry.
